# Event and Comment

**Published:** 1915-10

**Authors:** 


### DENTAL REGISTER

Vol. LXIX OCTOBER, 1915 No. 10

### EVENT AND COMMENT

GREEN VARDMAN BLACK. Another friend, and another of our great men has gone from us, and while we mourn the departure of our esteemed friend, we rejoice that it was our good fortune to have known and loved so good and great a man and to have had the benefit of his inspiring personality to stimulate our duller perceptions of duty and opportunity. It would seem superfluous for us to attempt to enumerate in any comprehensive manner the tremendous works accomplished by this great man; or to estimate with tolerable accuracy the tremendous usefulness of his life for the present and generations as yet unborn; because of the fundamental precepts that he has taught us to respect and which he has sent out through innumerable students and practitioners whom he has influenced, and by his writings, all to be projected by them into future ages. Dr. Black, from boyhood days, was curious to know the truth about things which came under his observation, and he became a student and remained one all his active life. Without a college education he became a scholar, and through his own personal endeavors and indomitable will he accomplished what most men believe the impossible. Like others, the more he learned the greater became his thirst for knowledge, and with such a spirit in his heart he determined to know all he could about the environments in which he found himself. It is fortunate that the necessities of life led him to select dentistry as his life work, because this turned his great mind and purpose in life into a chanel which gave him a broad and versatile career of investigation, which happily captivated his affections, until he became willing to give his life and all its energies to the calling for which he was to ultimately lay down his very life. We are apt to think that when one does so much for any one cause that he has no other ideals or concerns in life. Those who knew Dr. Black well will testify that his was not a one-sided life. He was a student of natural history, of science, literature, political economy, art, and the humanities in general, and these subjects were not superficially but profoundly studied by him with almost the same earnestness of purpose he was accustomed to approach his more technical and professional problems. The illustrations in his book were drawn by himself, he was a master of mechanical drawing. He also once told us he had in mind, when the opportunity came, to write a novel. We predict that it would have had more of truth than the usual productions of our present writers of fiction. It was this rare combination of attributes that made Dr. Black the interesting and forceful man he was in whatever company he found himself. He was fond of sports of all kinds and could enjoy and hold his own in repartee and anecdote with the best company. As a teacher he was beloved for his earnestness and endeavors to lead his students to higher attainments and respected for the strength of his convictions. With all his ability and accomplishments he was a man of reserve, in so far as he was personally concerned. He was never a politician or a fixer of professional affairs, and yet some of the great professional advancements were first suggested by him. He was the first to suggest the organization of dental teachers into an association for mutual improvement, which today is one of the most useful organizations we have. It was he who first suggested and began during his presidency, the reorganization of the National Dental Association, on its present basis, and many other such affairs for the good of organized professionalism were devised or ably supported by him. Now just what can we say as to the value of such a life to the profession for which he was devoted, or to the general public which has received through his activities the great benefits which no one can number? He was not only a pioneer in technical and scientific dentistry of the modern type, but he taught us the value of dentistry as a branch of the great healing art. His life, therefore, was a tremendous source of blessing to all humanity, as he gave unstintedly of his learning and skill as a student, practitioner and instructor. No man has or could have done more for the profession of dentistry, and his name and works will endure.

